# Overexpression of the *p53*-inducible brain-specific angiogenesis inhibitor 1 suppresses efficiently tumour angiogenesis

**DOI:** 10.1038/sj.bjc.6600067

**Published:** 2002-02-01

**Authors:** D G Duda, M Sunamura, L Lozonschi, T Yokoyama, T Yatsuoka, F Motoi, A Horii, K Tani, S Asano, Y Nakamura, S Matsuno

**Affiliations:** First Department of Surgery, Tohoku University Medical School, 1-1 Seiryomachi, Aoba-ku, 980-8574 Sendai, Japan; Department of Molecular Pathology, Tohoku University Medical School, 1-1 Seiryomachi, Aoba-ku, 980-8574 Sendai, Japan; Department of Hepatology/Oncology, Institute of Medical Science, University of Tokyo, 108-8639, Japan; Laboratory of Molecular Medicine, Institute of Medical Science, University of Tokyo, 108-8639, Japan

**Keywords:** angiogenesis, tumour, p53, brain-specific angiogenesis inhibitor 1

## Abstract

The *brain-specific angiogenesis inhibitor 1* gene has been isolated in an attempt to find fragments with p53 “functional” binding sites. As reported herein and by others, brain-specific angiogenesis inhibitor 1 expression is present in some normal tissues, but is reduced or lost in tumour tissues. Such data and its particular structure prompted the hypothesis that brain-specific angiogenesis inhibitor 1 may act as a mediator in the local angiogenesis balance. We herein demonstrate that brain-specific angiogenesis inhibitor 1 over-expression suppresses tumour angiogenesis, delaying significantly the human tumour growth in immunodeficient mice. The inhibitory effect of brain-specific angiogenesis inhibitor 1 was documented using our intravital microscopy system, strongly implicating brain-specific angiogenesis inhibitor 1 as a mediator in the control of tumour angiogenesis. In contrast, *in vitro* tumour cell proliferation was not inhibited by brain-specific angiogenesis inhibitor 1 transfection, whereas some level of cytotoxicity was assessed for endothelial cells. Immunohistochemical analysis of tumour samples confirmed a reduction in the microvessel density index in brain-specific angiogenesis inhibitor 1-overexpressing tumours. At messenger level, moderate changes could be detected, involving the down-regulation of vascular endothelial growth factor and collagenase-1 expression. Furthermore, brain-specific angiogenesis inhibitor 1 expression that was lost in a selection of human cancer cell lines could be restored by wild-type p53 adenoviral transfection. Brain-specific angiogenesis inhibitor 1 should be considered for gene therapy and development of efficient drugs based on endogenous antiangiogenic molecules.

*British Journal of Cancer* (2002) **86**, 490–496. DOI: 10.1038/sj/bjc/6600067 www.bjcancer.com

© 2002 The Cancer Research Campaign

## 

In recent years, the work of many cancer research groups has been oriented toward the understanding of the events that take place at the cellular and molecular level associated with the process of angiogenesis. It has been widely accepted that tumour growth and progression are dependent on angiogenesis (Folkman, 1971), a process in which numerous factors have been found to be involved. Moreover, recent evidence points toward the implication of genetic alterations commonly present in pancreatic cancer, such as those involving *p16* or *p53*, in the ‘switching’ on of angiogenic phenotypes (Harada *et al*, 1999; Nishizaki *et al*, 1999). For example, transduction of wild-type *p53* in a mutant *p53*-expressing human lung cancer cell via a recombinant adenovirus vector was demonstrated to reduce the tumour growth *in vivo*, in part by a bystander effect mediated by the antiangiogenesis mechanism (Nishizaki *et al*, 1999). This effect has been attributed to the ability of *p53* to reduce the expression of the endogenous angiogenic factor, vascular endothelial growth factor (VEGF) and enhance the expression of a newly described antiangiogenic factor, brain-specific angiogenesis inhibitor – BAI1 (Nishimori *et al*, 1997). Several other mechanisms affecting tumour angiogenesis have also been presumed, such as the induction of maspin, a member of serpin family (Zhang *et al*, 2000; Zou *et al*, 2000), and of thrombospondin 1 (Demeron *et al*, 1994). *BAI1* has been isolated and characterized by Nishimori and co-workers as a gene that is transactivated by p53. It has been proposed to be a member of a novel family of seven-span transmembrane receptors that also includes BAI2 and BAI3, cloned and described by Shiratsuchi *et al* (1997). The extracellular region of BAI1 uniquely encompasses five thrombospondin type I repeats and also one RGD (Arg-Gly-Asp) motif, which can be recognized by integrins. Due to its structure, BAI1 has been considered as a inhibitor of angiogenesis and a candidate for the glioma-derived angiogenesis inhibitory factor (GD-AIF), previously described by Van Meir *et al* (1994). Furthermore, in subsequent reports, BAI1 has been found to be absent or significantly reduced in glioblastomas (Nishimori *et al*, 1997), but also in many colorectal (Fukushima *et al*, 1998; Yoshida *et al*, 1999), breast cancers (Nishizaki *et al*, 1999), and lung cancers (Hatanaka *et al*, 2000), as compared to the extraneoplastic tissues. This suggests that in these tumours there is a correlation between the loss of its expression and the angiogenic response. Although the *BAI1* gene structure and associated proteins have been comprehensively described (Nishimori *et al*, 1997; Shiratsuchi *et al*, 1998a,b; Oda *et al*, 1999b), the mechanism of its antiangiogenesis effect is still largely uncharacterized. Previously, a recombinant protein corresponding to the extracellular region of BAI1 has been reported to inhibit angiogenesis that was experimentally induced by basic FGF in rat cornea (Nishimori *et al*, 1997). Moreover, a recombinant protein corresponding to the TSP-1 type 1 repeats was found to be a strongly antiangiogenic (Tolsma *et al*, 1993). Taken together, these results lead to the presumption that BAI1 may be a mediator in the p53-signalling pathway and to our hypothesis that BAI1 could be used to efficiently target the tumour-related angiogenesis.

Considering all the above-mentioned reports, in an attempt to extend the study of the BAI1 effects on human tumours, we checked the expression of this gene in pancreatic and colon cancer. In addition, we have investigated the interrelation between the presence of BAI1 and human pancreatic tumour development, associated very frequently with *p53* mutation. To this end, BAI1 expression was first checked the in nine human pancreatic cell lines, all of which present the loss of *p53* integrity, and also in tumour tissue samples from 14 patients. Furthermore, by means of RT–PCR, we evaluated the changes in proteolytic balance and endothelial cell mitogens incurred by the *BAI1* gene transfection in tumour cells. For the *in vivo* models, we transfected BAI1 or the *LacZ* gene into a human pancreatic adenocarcinoma cell line (Panc-1) by means of adenoviral-mediated transfer. Subcutaneous and skin chamber growth of wild-type and transfected tumour cells were observed and measured. We have analyzed visually the early human tumour angiogenesis with our transparent chamber model in immunodeficient mice. In short, this is the first report detailing the inhibitory effect of BAI1 on a human tumour as applied by gene therapy.

## MATERIALS AND METHODS

### Cell lines and transfections

In order to check the expression of *BAI1* we used six well-known human pancreatic adenocarcinoma cell lines (Panc-1, MiaPaCa2, AsPC-1, BxPC3, PCI35 and Su.Su.86), three cell lines established in our department (PK-1, PK-8, and PK-9, Kobari *et al*, 1986), and two widely-known colon cancer cell lines (T-84 and HT-29). Panc-1, MiaPaCa2, and T-84 cells were maintained in DMEM (Gibco, Tokyo, Japan) with glucose, the others in RPMI 1640 (Gibco, Tokyo, Japan), except for HT-29 cells that were cultured in McCoy's 5 A medium (Gibco, Tokyo, Japan). Finally, human umbilical vein endothelial cells (HUVEC), cultured in 200S medium (Kurabo, Tokyo, Japan) were used for *in vitro* experiments. All culture media were supplemented with 10% foetal calf serum, 100 U ml^−1^ penicillin and 0.1 mg ml^−1^ streptomycin and cells were kept in a humidified 5% CO_2_ atmosphere at 37°C. Gene transfer in Panc-1 cells was achieved using replication-deficient recombinant adenoviral vectors encoding the full length cDNA of *BAI1*, *p53* or *LacZ*, at a multiplicity of infection (MOI) of 10 for 36 h, as described (Nishizaki *et al*, 1999). The *p53* transfection was performed also for two colon cancer cell lines. HUVECs were infected with adenoviruses encoding *BAI1* or *LacZ*. The reporter gene transfer efficiency was assessed using beta-galactosidase staining, described elsewhere (Jiao *et al*, 1993).

### Mice

Severe combined immunodeficient mice (Fox Chase C.B-17/Icr-SCID Jcl) 6–8-weeks-old were used in all experiments, and kept in pathogen free conditions. Mice bearing dorsal skinfold chambers were housed individually. This study was approved by the Ethical Committee of Tohoku University Graduate School of Medicine. We observed the United Kingdom Co-ordinating Committee on Cancer Research (UKCCCR) guidelines for the welfare of animals in experimental neoplasia.

### *In vitro* and *in vivo* growth measurement

In order to evaluate the cytotoxic effects of *BAI1* expression on Panc-1 cells and on HUVECs, we performed MTT assays. One thousand tumour cells were plated in a 96-well plate and infected for 36 h with adenoviral vector encoding *BAI1* using multiplicities of infection from 0–200 (16 wells for each of the MOI used). Media was changed after 36 h, and 5 days after infection, 10 μl of sterile tetrazolium salt, MTT (3[4,5-dimethylthiazol-2-yl]-2,5-diphenyl-tetrazolium bromide, Sigma Chemical Co, St Louis, MO, USA) was added to the wells and incubated for 4 h at 37°C. Finally, 100 μl of 10% SDS were added, and after incubation at 37°C overnight, the plate was read at 490 nm. The *in vitro* growth of 2×10^5^ of the Panc-1 transfectants (MOI 10) used for *in vivo* experiments was compared to wild-type cells by crystal violet (1%) staining at day 6.

Subcutaneous growth curves in NK-depleted SCID mice of Panc-1 wild-type, Panc-1/BAI1, and Panc-1/LacZ (1×10^6^ cells, *n*=5) were assessed by measuring the tumour volume using a calliper. Tumour volumes were estimated by the following formula: *V*=*D*×*d^2^*×*0.4* (*V*=tumour volume *D*=largest dimension, and *d*=smallest dimension).

### Dorsal skin chamber model

Transparent chamber and *in vivo* microscopy system are reliable means for evaluation of tumour angiogenesis (Lehr *et al*, 1993). We used a simplified system (Duda *et al*, 2000) designed by us, and manufactured by Aoba Science Ltd. (Sendai, Japan). Tumour angiogenesis following implantation of wild-type Panc-1 or transfectants (1×10^6^ cells) was observed biweekly for 24 days using a Optiphoto 066 microscope (Nikon, Tokyo, Japan), a CCD camera (TEC-470 Optronics Co. Chelmsford, MA, USA) attached to the microscope, and the images were recorded on a video tape recorder (Victor, Tokyo, Japan). The recorded data were analyzed off-line.

### Semiquantitative RT*–*PCR

The expression of BAI1 in normal and tumour pancreatic tissue, and in pancreatic and colon cancer cell lines was assessed by RT–PCR. Total cellular RNA was extracted from a normal and 14 tumour pancreatic tissue samples, and from 1×10^6^ tumour cells using RNeasy Mini Kit (Qiagen KK, Tokyo, Japan), then 10 μg aliquots were reversed-transcribed using a RNease H Minus RT (Toyobo Co., Tokyo, Japan), and 1 μg of the resultant cDNA was amplified. Basic FGF, VEGF, MMP-1, ETS-1, and uPA were also amplified. The amplification of MSH2 was used as internal control. The specific amplification conditions are shown in Table 1

**Table 1:**
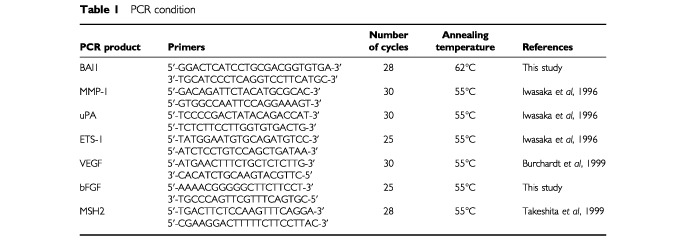
PCR condition

. Finally, aliquots of 10 μl from the amplification reactions were electrophoresed on 2% agarose (FMC BioProducts, Rokland, ME, USA) Tris-acetate EDTA gels and autoradiographed.

### Antibodies and histopathology

Rabbit anti-asialo GM1 antibody (Wako, Japan) was administered to SCID mice twice a week in order to block the non-specific immunity conferred by the NK cells. Standard haematoxylin and eosin staining was performed on 3–5 μm sections from paraffin-embedded tumour samples. Immunostaining of endothelial cells in tumour tissue was performed using rat anti-mouse PECAM-1 (CD31) antibody (BD Pharmingen, San Diego, CA, USA), as described (Vlaykova *et al*, 1997). Tissue sections (three per tumour) from control and transfectant tumours (*n*=5) were stained, and capillaries (diameter <10 μm) were counted in 10 high magnification fields per section.

### Statistical analysis

All experiments were performed in duplicate or triplicate. A two-tailed student *t*-test was used for statistical analysis of comparative data. Values of *P*<0.05 were considered significant.

## RESULTS

### Expression of BAI1 in tissue and in tumour cells

**Figure 1:**
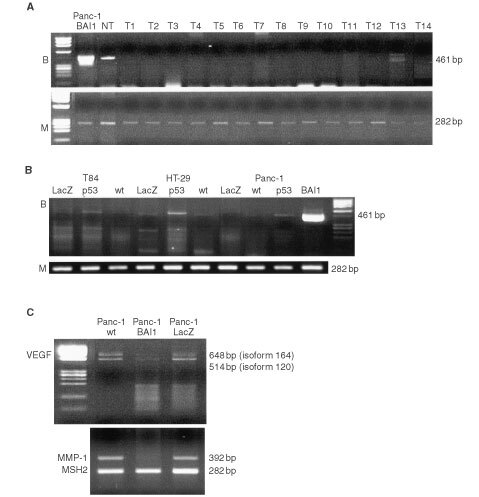
RT–PCR results showed that the expression of BAI1 that could be detected in normal pancreatic tissue (NT) was completely lost in all samples from pancreatic tumour tissue (T1 through T14) checked (**A**). On the other hand, strong BAI1 expression – designated B was confirmed after the adenovirus-mediated transfer. Interestingly, some level of expression could be restored in Panc-1 tumour cells, as well as in two colon carcinoma cells (HT-29 and T84) upon transfection of wild-type p53, but not after LacZ adenoviral transfer in these cells (**B**). Amplification for MSH-2 – designated M – was used as internal control in all experiments. RT–PCR for VEGF and a duplex reaction using primers for MMP-1 and MSH-2 indicated a reduction in MMP-1 expression level, and to a limited extent, of VEGF, in human pancreatic adenocarcinoma cells Panc-1 after BAI1, but not after LacZ transfer (**C**).

Of particular significance, as reported by others for colon and lung tissues, we detected BAI1 expression at messenger level in the normal pancreatic tissue, but not in any of the pancreatic and colon adenocarcinoma cell lines or tumour tissues checked (Figure 1A,B

). It is still unclear what type of cell is responsible for the expression in these normal tissues. In addition, there is no documentation as yet about the predicted existence of a soluble form of BAI1 upon cleavage of the extracellular region. A strong BAI1 expression could be confirmed by RT–PCR in Panc-1/BAI1 cells, but lacked completely in *LacZ*-transfected or wild-type Panc-1 cells. Interestingly, after *p53* adenoviral transfer, BAI1 expression was induced in Panc-1 and in the two colon cancer cell lines, T-84 and HT29 (Figure 1B), but not in the *LacZ* transfectants. On the other hand, the tumour cells used expressed an array of positive regulators of angiogenesis. Specifically, we identified by Northern blot and RT–PCR analysis the expression of several positive regulators of angiogenesis and invasion: endothelial growth factors- VEGF, bFGF, uPA, and a signal factor induced by the mitogens, ETS-1, respectively (unpublished data). The expression in tumour cells of some of these factors such as basic FGF, ETS-1, and uPA appeared not to be affected by transfer of either the *BAI1* or *LacZ* gene (not shown). However, BAI1 induced a decrease in VEGF level, as well as of that of interstitial collagenase 1 (MMP-1), the only metalloproteinase expressed in Panc-1 cells. This was not due to reporter gene transfection, as indicated by simple and duplex RT–PCR for MMP-1 and MSH2 (Figure 1C).

### *In vitro* tumour growth

**Figure 2:**
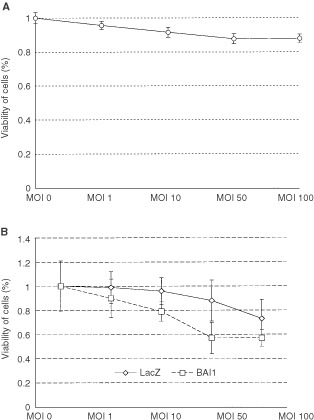
*In vitro* growth of tumour and endothelial cells after BAI1 transfection. The MTT assay was performed in duplicate, and for each MOI, an average of the optical density readings was calculated (±s.d., standard deviation). The reduction in optical density induced by adenoviral transfection was used as a measure of viability, normalized to cells incubated in the absence of transfection (MOI 0), which were considered 100% viable. Viability of cells at day 5 after transfection: (**A**) for Panc-1 tumour cells and (**B**) for HUVECs.

After 6 days of culture, there was no difference between the growth of parental Panc-1 cells and those of *BAI1* or *LacZ* transfectants. Crystal violet staining displayed a similar growth rate for Panc-1 cells and the Panc-1/BAI1 cells (MOI 10) used for *in vivo* studies (not shown). Moreover, beta-galactosidase staining of *LacZ* transfectants (MOI 10) confirmed a very high infection rate (almost 90%) of the tumour cells by the adenoviral-mediated transfer. Although no significant cytotoxic effect of BAI1 on tumour cells was observed by MTT assays, an antiproliferative effect after transfection into HUVECs was indicated (Figure 2

).

### *In vivo* tumour growth

**Figure 3:**
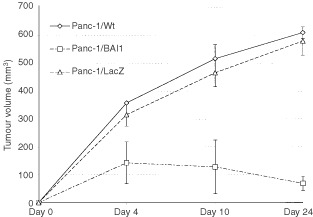
*In vivo* human tumour growth in NK-depleted SCID mouse. Measurements up to day 24 after subcutaneous inoculation demonstrated the very potent antiangiogenesis effect on tumours after BAI1 transfection, translated to a significant (*P<0.05) delay in tumour growth compared to the tumours in the wild-type and LacZ transfectant groups (average tumours volume±s.d.).

In contrast to wild-type Panc-1 and Panc-1/LacZ, which promoted a rapid tumour growth, as shown by tumour measurements up to day 24, the subcutaneous inoculation of Panc-1/BAI1 in immunodeficient mice significantly inhibited tumour growth (Figure 3

). After an initial onset of the tumour development, BAI1 presence was able to suppress angiogenesis and inhibited potently tumour growth. Despite the fact that expression of BAI1 *in vivo* after the adenovirus transfer was presumably lost in time, the tumour growth was delayed significantly even at day 84, when the mice were sacrificed, suggesting a bystander effect of BAI1. Although tumours were not cured, their growth was restricted by BAI1 over-expression following adenoviral transfection to an extent comparable to other strong angiogenesis inhibitors. There were no side effects apparent in any of the mice used throughout the experiments.

### *In vivo* microscopy

**Figure 4:**
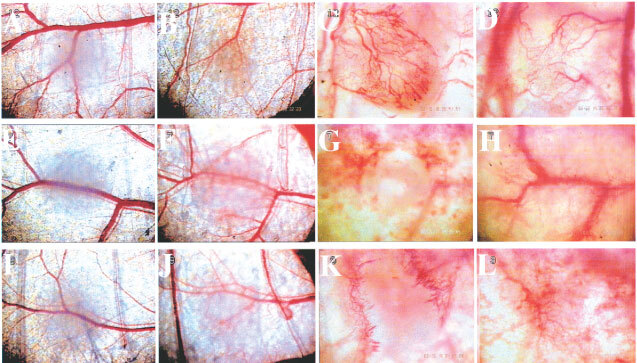
Tumour angiogenesis observation in transparent skin chamber after implantation of 2×10^5^ Panc-1 wild-type (upper panels), Panc-1/BAI1 (middle panels), or Panc-1/LacZ (lower panels) cells at day 0 (**A**,**E**,**I**), day 3 (**B**,**F**,**J**), day 7 (**C**,**G**,**K**) and day 14 (**D**,**H**,**L**). BAI1 transfected tumour cells failed to promote angiogenesis after 2 weeks, whereas the wild-type and LacZ transfected cells established a stable vascular network based on the normal vasculature of the skin, in a similar time frame.

Insights into the antiangiogenesis effect of BAI1 were gained by observation of tumour-related angiogenesis in our simplified transparent skin chamber model. Skin is a tissue with a high vascularity and thus, this system using immunodeficient mice and human tumour cells allows a very accurate assessment of tumour angiogenesis. In particular, vessel sprouting and the establishment of a tumour vessel network was observed surrounding and within the LacZ-transfected or wild-type Panc-1 cell implants as early as day 14 following cell implantation. In contrast, Panc-1/BAI1 cell implantation resulted in a significantly lower level of vascularity and the tumour appeared dormant after 2 weeks (Figure 4

).

### Histochemistry

**Figure 5:**
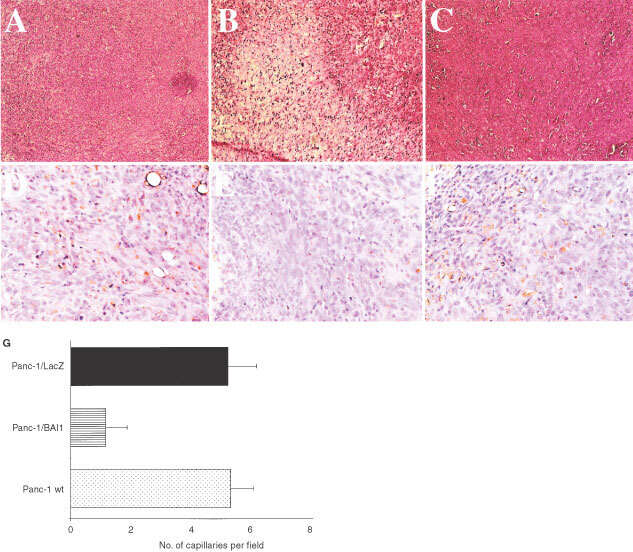
H&E staining of sections from Panc-1 (**A**), Panc-1/BAI11 (**B**), and Panc-1/LacZ 1 (**C**) tumours (magnification, ×10). Large areas of necrosis in the Panc-1/BAI1 tumour samples. Also, the expression of the endothelial cell marker CD31 in human Panc-1 (**D**), Panc-1/BAI11 (**E**), and Panc-1/LacZ (**F**) tumours from immunodeficient mice (magnification,×40) showed a decreased vascular index (**G**) in the BAI1 transfectant tumour samples.

Routine H&E staining revealed large areas of necrosis in the samples from Panc-1/BAI1 tumours as well as a significantly lower vascularity as compared to the Panc-1 wild-type and Panc-1/LacZ tumours after staining the tumour-related microvessels with PECAM-1 (Figure 5

). However, immunostaining showed no relevant difference in the expression of gelatinases (MMP-2 and MMP-9) between the three types of tumour (not shown).

## DISCUSSION

Tumour cells organize into solid tumours and metastasize due to a change from the normal phenotype in the local environment toward an angiogenic one. This is a consequence of the prevalence of proangiogenic factors compared to the antiangiogenic ones. We demonstrated by means of RT–PCR and Northern blotting that pancreatic and colon adenocarcinoma cells express various angiogenic factors like the endothelial cell mitogens- VEGF and bFGF, ETS-1 (documented as an angiogenic factor by Oda *et al*, 1999a), as well as haemoxygenase 1 (unpublished data), which has been recently found to induce angiogenesis (Deramaudt *et al*, 1998). The presence of these factors is likely to mediate the growth and invasion of these adenocarcinomas by induction of angiogenesis. On the other hand, the expression of angiogenic factors in tumours is paralleled by the decrease of the antiangiogenic factors. One of them, the recently discovered BAI1 has now been detected in normal pancreatic, colon and lung tissue, both at transcriptional and protein level, as reported previously and in this study. However, there was no detectable BAI1 expression in any of the *p53* defective pancreatic and colon cancer cell lines, nor in the pancreatic tumour tissue samples that were examined. Nevertheless, the wild-type 53 adenoviral-mediated transfer was able to restore the BAI1 expression in all tumour cell lines checked. These features displayed in pancreatic and colon cancer cells are in accordance with previous reports that the lack of BAI1 expression correlated with the *p53* mutation-related tumour angiogenesis. However, additional mechanisms seem to play a role in the regulation of BAI1, as a wild-type *p53* glioblastoma cell line – U87MG does not express BAI1 (Nishimori *et al*, 1997). Nevertheless, expression of BAI1 induced by adenoviral transfer in these cells did strongly inhibit their growth in the transparent skin chamber compared to parental cells, a feature that was not found after LacZ transfer (unpublished observations).

Several lines of evidence presented in this study emphasize the importance of the recently described *BAI1* gene. The presence of five thrombospondin type I repeats has been proposed as an explanation for its antiangiogenic effects, taking into consideration the effects on angiogenesis reported for other proteins containing these repeats such as TSP-1, TSP-2, METH-1, and METH-2 (Vazquez *et al*, 1999). Of particular interest is the fact that the *BAI1* gene transfer in tumour cells, similar to that of *p53*, seemed to change the RNA level of the endothelial cell mitogen VEGF, but did it alter other factors (bFGF, uPA, and ETS-1) implicated in the local angiogenic and proteolitic balance. Moreover, as reported for wild-type p53, BAI1 over-expression inhibited the expression of collagenase-1 (MMP-1), which is known to be a positive regulator of tumour angiogenesis (Sang, 1998), and was the only metalloproteinase we found to be expressed in Panc-1 cells. As yet, it is still unclear at what regulatory level this inhibition occurs, since BAI1 did not change the levels of ETS-1, which was implicated in the regulation of collagenase-1 and of other matrix metalloproteinases (Iwasaka *et al*, 1996).

*In vitro* experiments revealed that BAI1 transfection resulted in no detectable cytotoxic effect on tumour cells, but confirmed a level of cytotoxicity for endothelial cells, consistent with previous reports (Nishizaki *et al*, 1999). *In vivo*, subcutaneous and skin chamber implantation of Panc-1/BAI1 in NK-depleted SCID mice strongly suppressed tumour growth, while wild-type and *LacZ*-transfected tumours failed to do so. The immunostaining of tumour-related microvessels also showed a significant reduction of vessel density in Panc-1/BAI1, as compared to Panc-1 or Panc-1/Lac-Z tumour tissue. By capturing and off-line analysis of images from the transparent skin chamber, a decrease of vascularity in Panc-1/BAI1 tumour was directly observed and clearly indicated the inhibitory effect of BAI1 on angiogenesis.

Reported herein is also the observation that local overexpression of BAI1 disables any localized angiogenesis to such an extent that is able to induce an apparent state of dormancy in a transfected pancreatic adenocarcinoma cell line. Thus, even though the tumour cells were not killed, the tumour was unable to grow. One explanation for this phenomenon may involve an antiproliferative effect toward the endothelial cells, but the antitumour effects that occurs *in vivo* may be also due to putative interactions of the extracellular region of BAI1, since it is known to contain five thrombospondin type I repeats and of a RGD motif that is recognized by integrins. In addition, BAI1 inhibition of interstitial collagenase expression may prevent the dissolution of the extracellular matrix. Matrix metalloproteinase-1 (MMP-1) was found in other cells to be a *p53*-target gene, subject to p53 repression, mediated in part by associated protein 1 (Sun *et al*, 1999). We are currently investigating the MMP-1 protein level and activity in relation to BAI1 and p53 expression.

Taken together, this data offers further evidence that BAI1 is the candidate for the glioma-derived angiogenesis inhibitory factor (GD-AIF). It appears that the presence of the integrin-recognition motif RGD plays an important role in antiangiogenesis mechanisms and therefore we are currently investigating the effects of the transfer of BAI1 with a point mutation within the RGD motif.

The loss of BAI1 expression plays a role in the tumour angiogenesis-related cascade of events not only in brain-specific tumours, but also in other tumours types. These events are generally associated with the loss of p53 integrity or function. On the other hand, the overexpression of BAI1 inhibited strongly the pancreatic tumour-related angiogenesis. Therefore, since pancreatic carcinoma has a very poor prognosis after surgery, chemotherapy, and radiation therapy, BAI1 should be considered as a relevant candidate for future trials using this type of molecules or peptides derived from them, that would employ targeting of tumour angiogenesis as adjunctive or adjuvant therapy.
